# LGBTQ Older Adult Recruitment in the Midst of a COVID-19 Lockdown: Reminiscences of a Post-Doctoral Fellow

**DOI:** 10.1093/geroni/igab046.2245

**Published:** 2021-12-17

**Authors:** Robert Beringer

**Affiliations:** University of Victoria, Victoria, British Columbia, Canada

## Abstract

Within days of obtaining ethics approval for a qualitative study “Optimizing LGBTQ Engagement with Hospice and Palliative Care in the Island Health Region” our local Covid-19 lockdown began. It took several months to have new Covid-19 research protocols (Zoom Town Hall meetings/Zoom or telephone interviews) approved. Being impatient, I teamed with another group of researchers to launch “Covid-19: Your Current Experiences and Planning for the Future,” an online survey with a large qualitative component where we planned to oversample LGBTQ respondents. In time both projects were approved, and here I reflect on recruitment lessons learned. These include my perceptions how Zoom Town Hall meetings and interviews differ from those I’ve conducted in-person, reflections on how to use social media (including targeted Facebook advertising) to recruit participants, and sadly, how to manage anti-LGBTQ sentiment that resulted from even the most targeted advertising.

